# Development of Precipitation Hardening Parameters for High Strength Alloy AA 7068

**DOI:** 10.3390/ma13040918

**Published:** 2020-02-19

**Authors:** Julia Osten, Benjamin Milkereit, Michael Reich, Bin Yang, Armin Springer, Karina Nowak, Olaf Kessler

**Affiliations:** 1 Materials Science, Faculty of Mechanical Engineering and Marine Technology, University of Rostock, Justus-von-Liebig-Weg 2, 18059 Rostock, Germanybenjamin.milkereit@uni-rostock.de (B.M.); bin.yang@uni-rostock.de (B.Y.); olaf.kessler@uni-rostock.de (O.K.); 2 Competence Centre °CALOR, Department Life, Light & Matter, Faculty of Interdisciplinary Research, University of Rostock, Albert-Einstein-Str. 25, 18059 Rostock, Germany; 3 Electron Microscopic Centre, University Medical Centre Rostock, Strempelstraße 14, 18057 Rostock, Germany; armin.springer@med.uni-rostock.de; 4 Fraunhofer Research Institution for Large Structures in Production Engineering IGP, Albert Einstein-Str. 30, 18059 Rostock, Germany; karina.nowak@igp.fraunhofer.de

**Keywords:** AlZnMgCu, AA 7068, aluminium alloys, precipitation hardening, dilatometry, differential fast scanning calorimetry (DFSC), differential scanning calorimetry (DSC)

## Abstract

The mechanical properties after age hardening heat treatment and the kinetics of related phase transformations of high strength AlZnMgCu alloy AA 7068 were investigated. The experimental work includes differential scanning calorimetry (DSC), differential fast scanning calorimetry (DFSC), sophisticated differential dilatometry (DIL), scanning electron microscopy (SEM), as well as hardness and tensile tests. For the kinetic analysis of quench induced precipitation by dilatometry new metrological methods and evaluation procedures were established. Using DSC, dissolution behaviour during heating to solution annealing temperature was investigated. These experiments allowed for identification of the appropriate temperature and duration for the solution heat treatment. Continuous cooling experiments in DSC, DFSC, and DIL determined the kinetics of quench induced precipitation. DSC and DIL revealed several overlapping precipitation reactions. The critical cooling rate for a complete supersaturation of the solid solution has been identified to be 600 to 800 K/s. At slightly subcritical cooling rates quench induced precipitation results in a direct hardening effect resulting in a technological critical cooling rate of about 100 K/s, i.e., the hardness after ageing reaches a saturation level for cooling rates faster than 100 K/s. Maximum yield strength of above 600 MPa and tensile strength of up to 650 MPa were attained.

## 1. Introduction

AlZnMgCu alloys attain highest strength levels among aluminium alloys and are particularly used in aviation applications [1]. The strength is typically adjusted through precipitation hardening, respectively, age hardening [2,3]. The age hardening process comprises three major steps: Solution annealing, quenching, and ageing [1]. The solution heat treatment intends to dissolve relevant alloying element atoms and is setting the initial structural condition for the following cooling or quenching. Due to quenching the alloying element atoms shall be retained in solution resulting in a supersaturation. From this unstable state, nanometre sized precipitates form during the final ageing treatment, which hinder the dislocation glide and thereby increase the strength of the alloy. To obtain the intended properties, for instance high strength or sufficient ductility, a number of heat treatment parameters for the age hardening process must be carefully selected. This includes reasonable heating and cooling rates as well as temperatures and durations of the solution annealing and the ageing step. In terms of technological application the cooling step is critical as undesired quench induced precipitation during cooling might severely reduce the strength as well as the ductility after ageing [4,5,6]. Until the end of the last century, the process parameters of age hardening were predominantly acquired by empirical ex situ tests [1,7]. Nowadays the kinetics of quench induced precipitation can be investigated by in situ differential scanning calorimetry (DSC, e.g., [8]), as well as using in situ dilatometry (DIL) [9]. Conventional DSCs are limited to cooling rates of about 5 K/s. However, high strength aluminium alloys have critical cooling rates for a complete supersaturation of the solid solution of up to some hundreds of K/s. Basically DIL should allow the in situ cooling analysis of precipitation with cooling rates of up to about 100 K/s [9]. Thus, in this work, the DIL method is further developed in order to enlarge the cooling rate range and to enhance the sensitivity. Another sophisticated technique for the kinetic analysis of quench induced precipitation is the chip-sensor based differential fast scanning calorimetry (DFSC [10]) using a differential reheating method (DRM) [11].

This work intends to enable a sound selection of appropriate heat treatment parameters for the high alloyed aluminium alloy AA 7068 based on the kinetic analysis of the related solid-solid phase transformations. The major objective was adjusting a high strength to allow the usage of AA 7068 as a material for solid self-piercing rivets [12,13] applied for medium to high strength AlMgSi and AlCuMg sheet material [14]. Therefore, the kinetic behaviour of dissolution and precipitation reactions during heating, cooling, and annealing was investigated by in situ DSC as well as in situ differential DIL in a broad range of scanning rates and durations. In addition, the DFSC-DRM was used to reveal the critical cooling rate. Quench induced precipitations of slower cooling rates were investigated by SEM. The continuous cooling precipitation diagram of AA 7068 has been recorded. Moreover, the hardness and tensile properties of selected states including ageing/stretching after quenching were tested resulting in a complete set of suitable age hardening heat treatment parameters, which obtain high strength for AA 7068.

## 2. Materials and Methods

The investigated aluminium wrought alloy AA 7068 (AlZn7.5Mg2.5Cu2) was supplied as a rolled bar stock with a diameter of 8 mm. The mass fractions of alloying elements are given in Table 1 (analysed by optical emission spectrometry). This composition was chosen as it allows to adjust very high strength. It should be noted, that the Cu content is at the lowermost boundary, whereas the Zn content is slightly above the boundary. The initial heat treatment condition was T651, which is solution annealed, quenched, stress relieved by stretching, and artificially aged.

DSC was performed to analyse the kinetic dissolution and precipitation behaviour during heating of the initial conditions T651 and “as-quenched”. In particular, a temperature was determined which is suitable for solution annealing. Additionally, the kinetic behaviour of quench-induced precipitation was analysed via DSC. Two instruments were used, namely a Setaram 121 DSC (Setaram, Caluire-et-Cuire, France) and a Pyris Diamond DSC (PerkinElmer, Waltham, MA, USA). The DSC experiments were performed as described in references [15,16]. For each condition at least four samples and two baselines were measured.

In addition to DSC, the quench-induced precipitation was analysed by DIL, which basically allows detection of precipitation reaction in Al alloys [17,18,19], although their volume effect is very small. Beyond that it enables an extension of the accessible cooling rates [9]. In this work the dilatometric method has further been developed to enlarge the assessable cooling rate range and to increase the sensitivity for the detection of precipitation during cooling.

**Figure 1:**
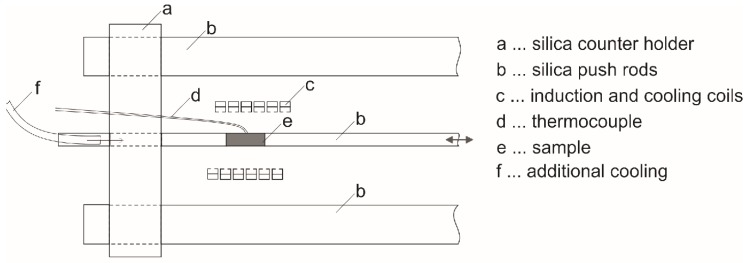
Schematic setup of the Bähr dilatometer in alpha-mode.

Dilatometric measurements were performed using a Bähr 805A/D instrument with a linear variable differential transducer sensor (LVDT), utilising silica push rods to transmit the change in length/elongation, see Figure 1.

**Figure 2:**
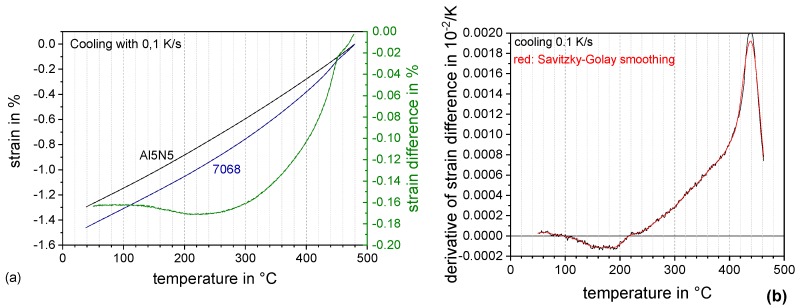
Evaluation procedure dilatometric analysis: Alloy 7068, solution annealing 480 °C 10 min, and cooling rate 0.1 K/s. (**a**) raw strain data of sample and baseline measurement as well as strain difference of these two and (**b**) derivation of strain difference.

The specimens (hollow cylinders in 4 mm diameter, 10 mm length, and thickness 1 mm or 0.2 mm) were inductively heated by an induction coil. An additional inner hollow coil is perforated and thus used for inert gas quenching. For cooling up to 10 K/s nitrogen and for more rapid cooling helium were used as inert and cooling gases. Hollow rods allow an internal cooling by slight gas volumes flow through hollow samples. The temperature of the sample was controlled by S-type thermocouples spot-welded to the sample surface. Heating to solution annealing at 480 °C for 10 min was carried out at 2 K/s. Cooling was realised at five different cooling rates, namely 0.1, 1, 10, 30, and 100 K/s. For each experiment, a fresh sample was used. The same heat treatments were done on 99.9995% pure Al (Al5N5) alternating with AA 7068 samples. It is expected that the Al5N5 samples only show the thermal expansion of the aluminium matrix together with the push rods and can therefore be used for baseline measurement. The baseline is new for dilatometry of Al alloys and allows to correct the signal for the basic expansion of the aluminium matrix lattice. Beyond that, it contains all additional influences of the device on the dilatometry result, e.g., additional length changes of the silica push rods. The strain *ε* is evaluated dividing the length change ΔL by the initial sample length *L_0_*, ε = ΔL/L_0_. For a reduction and a smoothing of raw data a stepwise linear description with 500 points between 50 and 478 °C has been used. The pure Al baseline strain *ε_BL_* was subtracted from the sample strain *ε_S_* (see Figure 2a) and the strain difference has been derived with respect to temperature (dΔε/dT). The derivative is further smoothed by the Savitzky–Golay [20] filter method of the software OriginPro 2018 which performs a local polynomial regression to determine the smoothed value for each temperature point. The resulting curve can finally be used for evaluation, e.g., of precipitation start and end temperatures (Figure 2b). As a consequence of the baseline subtraction, the zero-level of the derivative curve should now be the reference value (being zero), allowing easy determination of the characteristic temperatures. For each condition at least three samples and two baselines were measured by DIL. The dilatometric experiments in general showed a very good reproducibility. Hence, one representative curve of three cooling tests was used for further discussion.

As the in situ detection of precipitation during cooling by dilatometry is limited to cooling rates of 100 K/s, even faster cooling experiments were performed by Differential Fast Scanning Calorimetry (DFSC) applying the Differential Reheating Method (DRM) introduced in [11,21]. In addition, hardness testing (HV1) in the as-quenched state as well as in the artificially aged state were performed. Tensile testing was performed using round tensile specimen with a screw head, a diameter of 5 mm and a gauge length of 25 mm, in accordance with DIN 50125:2016 form B following the test method B out of DIN EN ISO 6892-1:2017. (Testing machine Z50, ZwickRoell GmbH & Co.KG, Ulm, Germany).

The microstructural changes caused by quench-induced precipitation were investigated by scanning electron microscopy (SEM). Samples for SEM and energy dispersive X-ray spectroscopy (EDS) analysis were cold embedded in epoxy resin and prepared by standard grinding and polishing with water-free, ethanol-based lubricants [22]. For final polishing, a 0.05 µm oxide polishing suspension was used. The embedded and polished samples were mounted on a SEM carrier with adhesive conductive carbon tape (Co. PLANO, Wetzlar, Germany). Additionally, the SEM samples were coated with carbon (Leica EM SCD 500, Co. Leica, Wetzlar, Germany). SEM samples were analysed by field emission SEM (MERLIN^®^ VP Compact, Co. Zeiss, Oberkochen, Germany) equipped with an EDS detector (XFlash 6130, Co. Bruker, Berlin, Germany). For SEM imaging, an Everhart–Thornley-type high-efficiency secondary electron detector (Co. Zeiss, Oberkochen, Germany) was used, applying an accelerating voltage of 5 kV. The particle size was determined with the Imagic ims Client software. Representative areas of the samples were analysed and mapped for elemental distribution on the basis of the EDS spectra obtained at acceleration voltages of up to 20 kV and evaluated by the QUANTAX ESPRIT Microanalysis software (version 2.0, Co. Bruker, Berlin, Germany).

**Figure 3:**
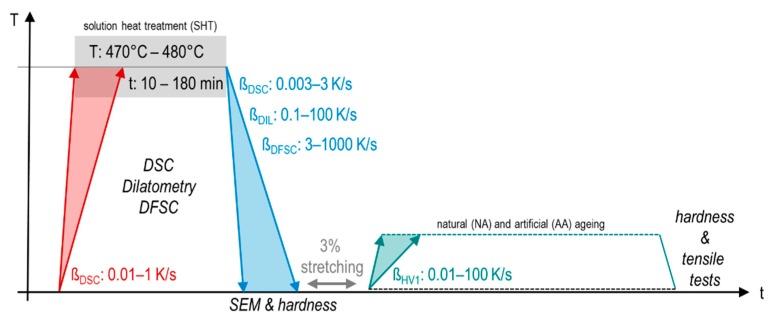
Schematic temperature-time profile.

The different analysis techniques used in correlation with the particular applied heat treatments are illustrated in Figure 3.

## 3. Results and Discussion

### 3.1. DSC Heating Curves: Determination of an Appropriate Solution Annealing Temperature

**Figure 4:**
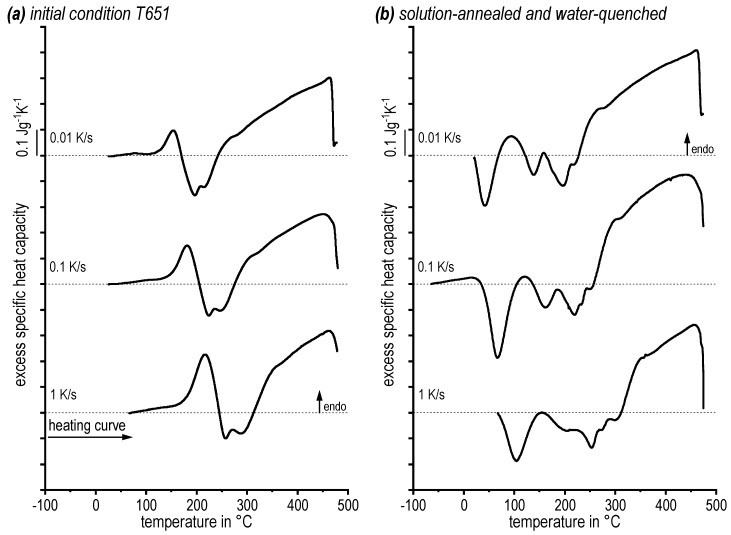
Differential scanning calorimetry (DSC) heating curves of alloy 7068: (**a**) initial condition T651 and (**b**) solution-annealed and water-quenched.

The sequence of endothermic dissolution and exothermic precipitation reactions during heating of alloy 7068 has been analysed for various heating rates. Previous DSC melting tests with 0.3 K/s revealed the solidus temperature to be 491 °C [23]. Thus, the temperature range analysed extends from 20 °C (sometimes from −70 °C) up to 485 °C, keeping the sample in the solid state. For improved readability the DSC curves shown in Figure 4 are shifted, showing the curve with the lowest heating rate on top. Endothermic changes are plotted upwards. The dissolution behaviour of two different initial conditions, namely T651 (Figure 4a) and as-quenched (Figure 4b), was investigated.

The heating DSC curves reveal a complex dissolution and precipitation behaviour with alternating endo and exothermic regions. The single reactions severely overlap, making a deconvolution of single reactions difficult. However, between the two different initial conditions substantial differences are seen particularly within the temperature region below 300 °C. The T651 state mainly shows dissolution reactions of the initial aged state (precursors of η-Zn_2_Mg, S-Al_2_CuMg, etc.). The quenched state shows alternating precipitation and dissolution reactions according to the precipitation sequence of AlZnMgCu alloys (also precursors of η-Zn_2_Mg, S-Al_2_CuMg, etc.). Above about 300 °C dissolution processes of more stable precipitates (η-Zn_2_Mg, S-Al_2_CuMg, etc.) dominate in both cases. A suitable solution annealing temperature can therefore be determined from the slow warming DSC curves of 0.01 K/s, since the DSC signal of the main solution process drops abruptly towards zero at high temperatures of 472 °C. This means, the dissolution process is finished, and the heating rate specific solvus temperature is about 472 °C. It can therefore be assumed that an equilibrium solvus temperature is slightly below 472 °C. In the next step the influence of soaking within the temperature range between solvus and solidus temperature was assessed by subsequent DSC cooling curves.

### 3.2. DSC Cooling Curves: Assessing the Influence of Solution Annealing Temperature and Duration

**Figure 5:**
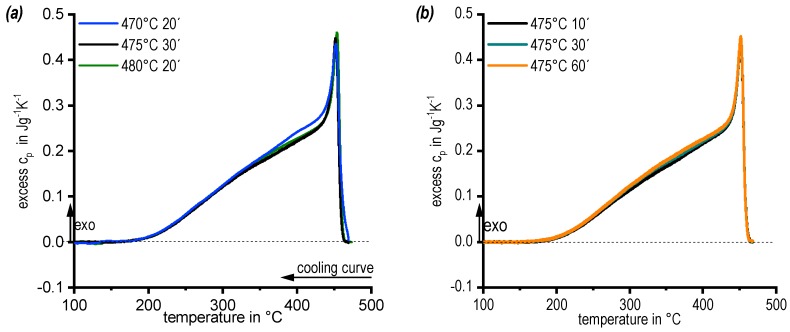
DSC cooling curves of alloy 7068 (cooling rate 0.01 K/s): (**a**) different solution annealing temperatures and (**b**) different solution annealing times.

To allow a sound selection of the solution annealing temperature and duration, DSC cooling curves after solution treatment at 470, 475, and 480 °C were compared, see Figure 5a. In general, the DSC cooling curves are very similar. They show at least two overlapping precipitation reactions (details are given in Chapter 3.3.). However, in detail it can be seen that after solution treatment at 470 °C for 20 min, an instantaneous onset of precipitation with onset of cooling occurs. This indicates an incomplete dissolution of the main alloying elements, acting as nuclei for precipitation [24]. To ensure a complete solution, the temperature for the solution treatment was set at 475 °C (−480 °C). In the next step the soaking duration was varied between 10 and 60 min, see Figure 5b. The DSC cooling curves from any soaking duration were highly similar. 30 min at 475 °C (10 min at 480 °C) were defined as solution treatment duration.

### 3.3. Kinetic Behaviour of Quench Induced Precipitation Analysed by DSC, DIL, DFSC, and Hardness Testing

**Figure 6:**
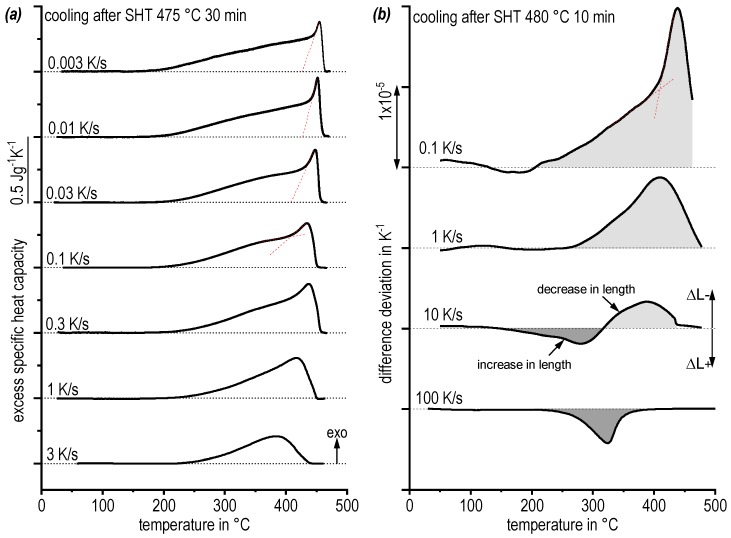
Cooling curves of alloy 7068 after solution annealing: (**a**) DSC and (**b**) differential dilatometry.

The kinetic behaviour of quench-induced precipitation during cooling with various rates from the solution annealing temperature has been analysed by DSC (Figure 6a) and dilatometry (Figure 6b). The cooling curves in Figure 6 are shifted and the slowest rates are shown on top. Be aware of the different rates used in DSC and dilatometry.

The DSC cooling curves show exothermic precipitation when the signal exceeds the zero level, since only exothermic precipitation reactions can occur during cooling. DSC cooling (Figure 6a) reveals a relatively sharp onset after slight undercooling (below the solvus) followed by a very broad reaction shoulder, similar to other highly concentrated AlZnMgCu alloys [8,25]. This undercooling is in the range of few K at the slow rate of 0.01 K/s and increases with increasing cooling rate. At the rate of 3 K/s the onset of precipitation is seen at about 440 °C and thus the undercooling is about 30 K. The second broad exothermic precipitation during slow cooling ranges from about 465 to 180 °C. This indicates a severe overlap of multiple exothermic precipitation reactions. With an increasing cooling rate, the peak area decreases, i.e., in total the precipitation reactions are supressed. The critical cooling rate cannot be quantified by conventional DSC measurement, since at the fastest rate of 3 K/s a precipitation enthalpy of about 20 J/g is still measured.

Differential dilatometry has been found to be useful for studying higher cooling rates of up to 100 K/s and thus can extend the bandwidth of analysed cooling rates. Figure 6b compares cooling dilatometry curves in the range of 0.1 to 100 K/s. A relative shortening of the sample is plotted upwards. It has been described earlier [9] that certain precipitation reactions cause an increase in the total volume and thus an elongation of the sample, while precipitation of a different phase might cause a shrinkage of the volume and therefore a shortening of the sample. That means, opposing volume changes caused by precipitation of different phases might superimpose each other and a signal value of zero does not necessarily mean that no reaction is occurring. Therefore cooling dilatometry bears the same issue like heating DSC in terms of its signal interpretation [16].

For cooling of aluminium alloy 7068, dilatometry reveals very similar results compared to DSC. For instance, comparing the cooling curves of 0.1 K/s, reveals a sharp onset of precipitation resulting in a peak of about 435 °C in both cases. Also, for the inflection towards the shoulder 410 °C are revealed as characteristic temperature in both cases. For 1 K/s the general curve shapes are similar, too. In addition, dilatometry helps to discuss temperature regions of overlapping reactions. The latter is observed at a cooling rate of 10 K/s where the dilatometer reveals a first precipitation region between about 440 and 320 °C causing a shortening of the sample. This is followed by another reaction region between about 320 to 150 °C, causing another elongation.

As a very positive aspect cooling dilatometry helps to substantially expand the cooling rate spectrum up to 100 K/s. For high alloyed AlZnMgCu alloy AA 7068 even this fast cooling rate of 100 K/s reveals a precipitation signal between about 380 and 220 °C. This finding shows that the upper critical cooling rate is above 100 K/s. Faster cooling of hollow samples may be possible in quenching dilatometry. However, the decreasing stiffness with deceasing wall thickness complicates length measurements. Thus, fast scanning calorimetry (DFSC) and hardness testing after cooling as well as after cooling and artificial ageing (130 °C for 16 h) are used to identify the upper critical cooling rate.

**Figure 7:**
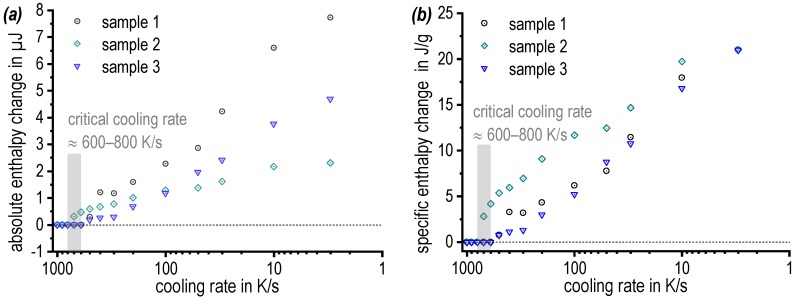
Differential fast scanning calorimetry (DFSC) results of three samples 7068: (**a**) absolute enthalpy change and (**b**) specific enthalpy change.

Figure 7 shows the results obtained by the DFSC-DRM method. Three individual samples were used to determine the precipitation enthalpy during cooling at various rates. One issue of the used method is, that the samples are very small with only about 100 by 100 by 40 µm^3^. Currently no device is available which allows to determine these samples’ mass. Thus, the normalization of the calorimetric signal, which is normally carried out via the sample mass, has to be based on assumptions. In this work, we were able to measure the cooling rate of 3 K/s in both, DSC and DFSC. We therefore used the value of the specific precipitation enthalpy obtained by DSC (integral of the normalised DSC curve, average value = 21 J/g) to calibrate the specific precipitation enthalpies measured by DFSC. This is the raw DFSC signal of each sample in µJ as shown in Figure 7a divided by the specific precipitation enthalpy of 21 J/g obtained by DSC to estimate the individual DFSC sample masses. With these estimated masses and the cooling rates applied, the DFSC signals are normalised and the values plotted in Figure 7b are obtained. Figure 7 shows substantial deviations between the three samples. Those deviations are related to the tiny sample size and probably originate from slight variation in the chemical composition (by local segregations) or from variations in the density of nucleation sites (dispersoid distribution and particularly the amount of grain boundary surface within the samples). Nevertheless, all samples show a similar trend and the specific precipitation enthalpy is dropping towards zero with increasing cooling rate. The upper critical cooling rate was found to be about 600 K/s to 800 K/s, which allows a complete supersaturation of the alloy. This is one of the highest critical cooling rates ever found for an aluminium alloy, compare [11,21,25,26].

**Figure 8:**
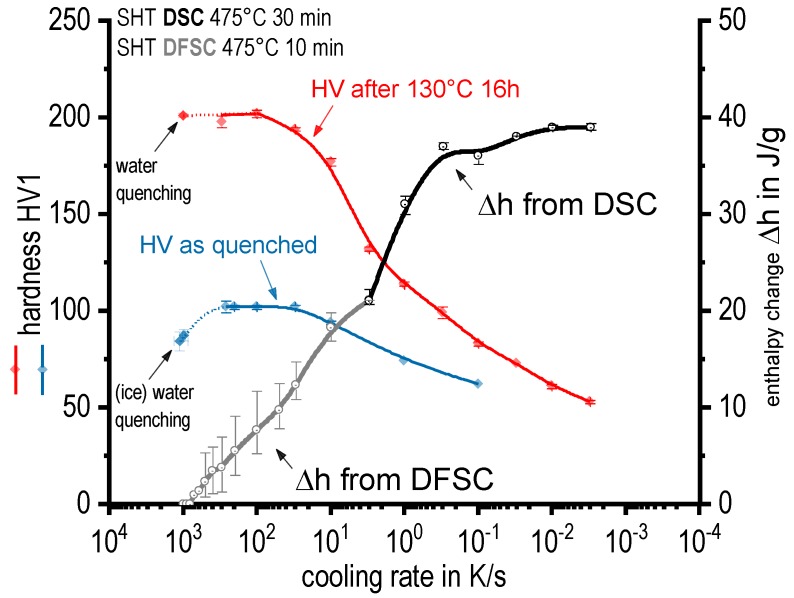
Enthalpy change of alloy 7068 from quench induced precipitation as well as hardness in as-quenched and aged condition.

**Figure 9:**
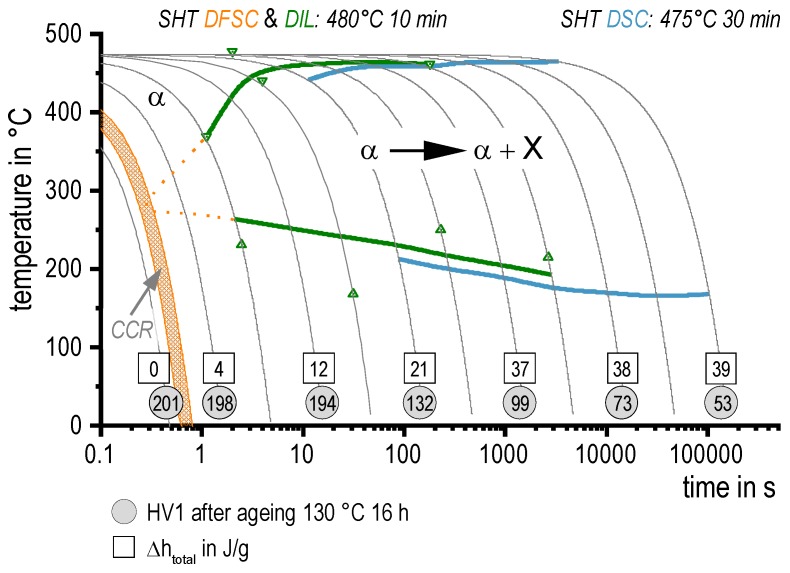
Continuous Cooling Precipitation Diagram of AA 7068 from DSC, dilatometry (DIL), and DFSC. Within the reaction α → α + X, the X comprises any quench induced phase.

To compare the precipitation enthalpies from DSC and DFSC with hardness values of the distinct cooling rates Figure 8 is plotted. Average, minimum, and maximum values are shown for the enthalpies. The averaged hardness values are plotted with their standard deviations from six single indentations. The cooling rate axis is scaled logarithmic with high cooling rates on the left in order to allow alignment with the time axis of the following continuous cooling precipitation diagram in Figure 9.

The specific precipitation enthalpies determined for slow cooling rates are quite high (up to 40 J/g), which is reasonable for this high concentrated alloy. With increasing cooling rate, the total precipitation enthalpy is decreasing as precipitation is suppressed. This behaviour is common for age hardening aluminium alloys [25]. As indicated above the upper critical cooling rate, which is the slowest cooling rate at which any quench-induced precipitation is supressed completely, is about 600 to 800 K/s. This is the highest value ever reported for an aluminium wrought alloy, which is reasonable, as AA 7068 is a quite high concentrated alloy. However, the hardness after ageing indicates a technical critical cooling rate to be just about 100 K/s. This means, that after artificial ageing (130 °C for 16 h) there is no further increase in hardness with an increasing cooling rate above 100 K/s. This can be attributed to a certain direct hardening effect of quench induced precipitation for cooling rates above 100 K/s. This indeed is confirmed by the as-quenched hardness, which first rises with increasing cooling rate, then shows a maximum/plateau for cooling rates between about 30 to 300 K/s. For even faster cooling rates the as-quenched hardness finally drops again when approaching the physical upper critical cooling rate. A direct hardening contribution of quench-induced precipitation has already been shown for alloy AA7150 [27]. In detail for AA7150 very similar hardness values in similar cooling rate regions were achieved. For AA7150 the direct hardening effect of quench induced precipitation was confirmed by tensile tests in the as-quenched condition and is primarily referred to the quench-induced precipitation of thin Zn and Cu rich Y-phase platelets [27,28].

All cooling results (DSC, DIL, DFSC, and hardness) are summarised in the continuous cooling precipitation (CCP) diagram of aluminium alloy 7068 in Figure 9, being a powerful tool for practical heat treatment as well as for heat treatment simulation [29].

### 3.4. Microstructure of Different Cooled Samples through Scanning Electron Microscopy (SEM)

**Figure 10:**
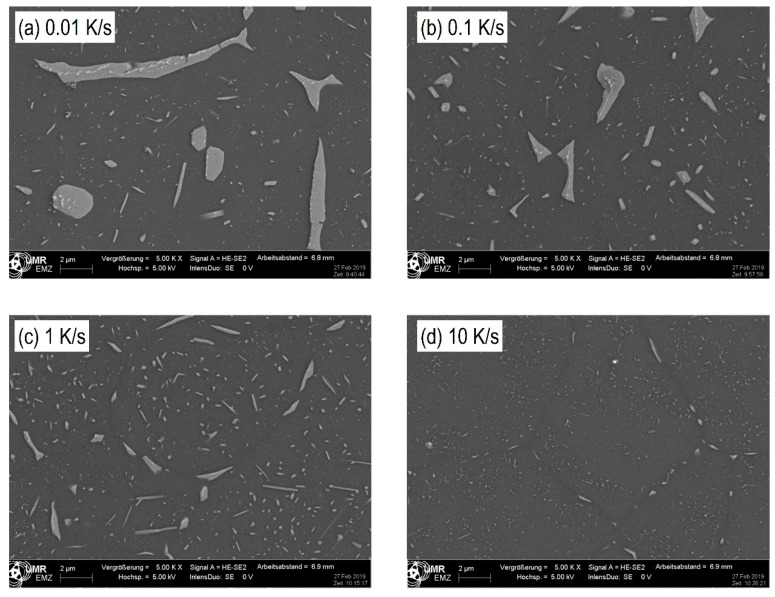
SEM-secondary electron images of alloy 7068: Cooling rates (**a**): 0.01 K/s; (**b**): 0.1 K/s; (**c**) 1 K/s; and (**d**) 10 K/s.

**Figure 11:**
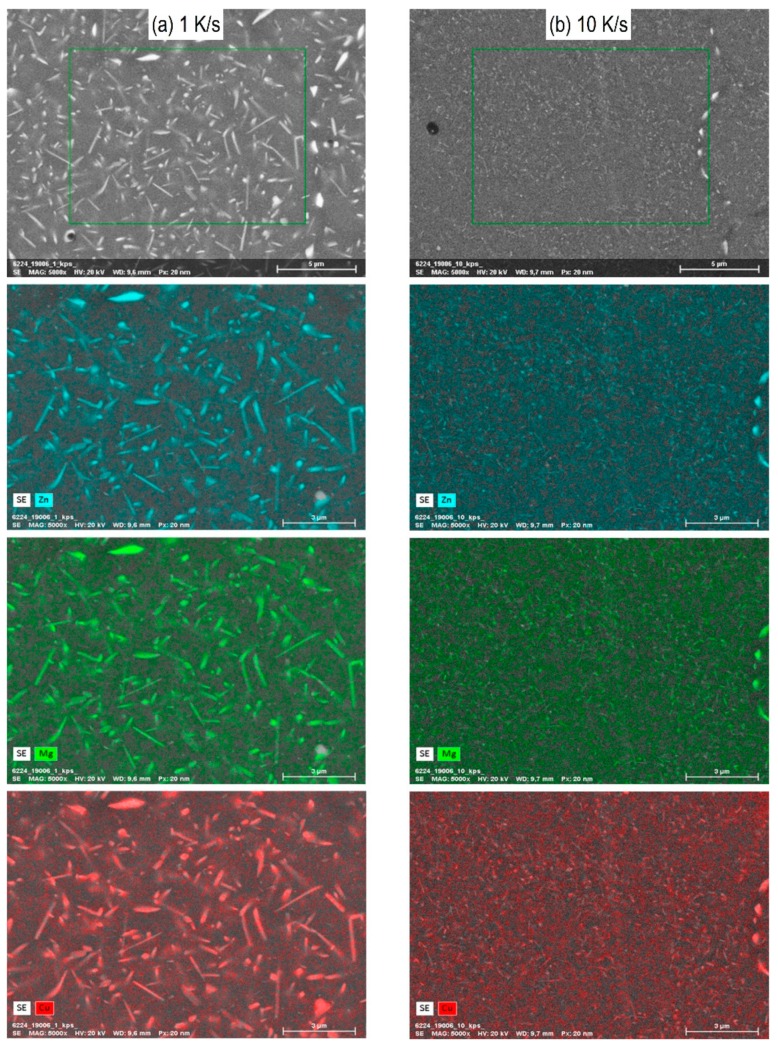
SEM-EDS maps for Zn, Mg, and Cu of alloy 7068 after cooling at (**a**) 1 K/s and (**b**) 10 K/s.

The microstructures of alloy 7068 after various cooling rates have been analysed by SEM-secondary electron images and selected images are shown in Figure 10 for cooling rates ranging from 0.01 to 10 K/s. Next to the aluminium matrix which appears dark, there are two main types of precipitates appearing brighter: Precipitates on grain boundaries and precipitates inside the grains. Particularly for the slower rates, the grain boundary particles become fairly large. Within those particles often even brighter particles are visible, which are probably Fe rich primary particles. Obviously, the quench induced particles become smaller with increasing cooling rate. This applies especially to the grain boundary precipitates. However, precipitates inside the grains from 0.01 to 1 K/s first appear to increase in size and particle density, but with further increasing rate these particles become substantially smaller. Within this work no detailed phase analysis was carried out. According to previous work precipitation of the following phases can be expected: S-Al_2_CuMg, η-Mg(Zn,Al,Cu)_2_, ZnCu rich Y-phase platelets [8,27,28,30], and potentially also precursor/cluster formation [31,32]. The latter two particle types are probably too small to be detectable by SEM. Thus, it seems likely that the majority of the particles observed in our SEM work are either S-Al_2_CuMg or η-Mg(Zn,Al,Cu)_2_ phase particles. EDS indicates an enrichment of Zn, Mg, and Cu in any quench induced particle observed in SEM (Figure 11) and thus does not allow to distinguish between the two phases. Within the CCP diagram (Figure 9) all quench induced precipitation reactions are summarised to as α → α + X.

### 3.5. Mechanical Properties after Ageing: Tensile Tests

**Figure 12:**
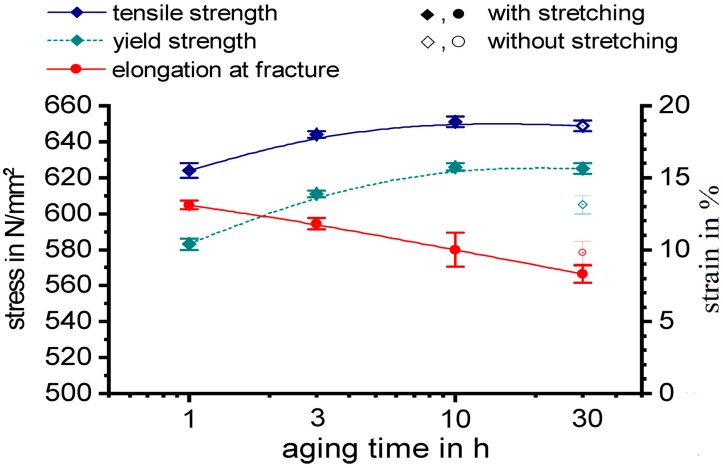
Mechanical properties of alloy 7068: With or without stretching 3%, different ageing times 1, 3, 10, and 30 h at 130 °C.

The tensile properties of aluminium alloy 7068 were tested for different ageing durations at 130 °C. Plain quenched samples were compared with samples which have seen an additional stretching of 3% after quenching and prior ageing, see Figure 12. For each condition six samples were tested. The results from tensile tests, yield strength R_p0,2_, tensile strength R_m_, and fracture elongation are summarised in Table 2 for T6 and T651 states. Next to the average values the standard deviations out of six tests are shown. Maximum yield strength and tensile strength were obtained for an ageing treatment at 130 °C for 10 h following on the stretching operation (T651). These strengths are slightly lower than for aluminium alloy 7068 in the T6511 state according to AMS4311. However, it must be considered that that the Cu content of the investigated batch of alloy 7068 is at the lowermost boundary (Table 1). Beyond that an additional stretching after aging (T6511) may result in a further increase in yield strength compared to T651. The fracture elongation is relatively high in all cases, while it is decreasing with increasing ageing duration.

## 4. Conclusions

Based on the experimental work comprising differential scanning calorimetry, differential dilatometry, differential fast scanning calorimetry, scanning electron microscopy, as well as hardness and tensile tests after age hardening heat treatment of a highly concentrated AlZnMgCu alloy AA 7068 the following findings can be stated:

- An appropriate temperature for solution treatment is determined to be 475 °C (−480 °C), as the heating rate specific solvus at 0.01 K/s is 470 °C;
- Cooling DSC after soaking durations of 10 to 60 min at 475 °C showed no differences, and a soaking duration of 30 min was chosen;
- The application of differential dilatometry allows the in-situ detection of quench induced precipitation in a wide cooling rate range of 0.1 K/s to 100 K/s. At fast cooling of 100 K/s dilatometry still reveals quenched induced precipitation;
- While DSC only shows one very broad precipitation peak for a wide range of cooling rates, dilatometry indicates the overlap of several quench induced precipitation reactions;
- The physical upper critical cooling rate (CCR) at which quench induced precipitation is fully suppressed was determined by DFSC analysis to be about 600 to 800 K/s;
- A technological CCR is found to be ≈ 100 K/s (i.e., hardness after artificial ageing remains on a saturation level for higher cooling rates including WQ). This indicates, that quench induced precipitation at cooling rates faster than 100 K/s contributes to a direct hardening effect;
- For the investigated batch of AA 7068 the age hardening treatment comprising of solution heat treatment 475 °C 30 min, water quenching, stretching 3%, and ageing (130 °C 10 h) results in a yield strength R_p0.2_ of 626 N/mm^2^, a tensile strength R_m_ of 651 N/mm^2^, and a fracture elongation of 10%.

## Figures and Tables

**Table 1:** Mass fraction of alloying elements of the investigated material in %.

Aluminium Alloy	Mass Fraction in %								
	Si	Fe	Cu	Mn	Mg	Cr	Zn	Ti	Zr
**AA 7068**	0.052	0.076	**1.60**	0.008	**2.96**	0.013	**8.43**	0.022	**0.084**
AMS 4331	≤0.12	≤0.15	**1.6–2.4**	≤0.10	**2.2–3.0**	≤0.05	**7.3–8.3**	≤0.10	**0.05–0.15**

**Table 2:** Parameters from tensile testing: Heat treatment with or without stretching.

	T6 475 °C 30 min, Quenching in Water, 130 °C 30 h	T651 475 °C 30 min, Quenching in Water, Stretching 3%, 130 °C 10 h
yield strength in N/mm^2^	605 ± 5	626 ± 2
tensile strength in N/mm^2^	649 ± 2	651 ± 3
elongation at fracture in %	9.8 ± 0.8	10.0 ± 1.2
